# Corona Virus (COVID-19) “Infodemic” and Emerging Issues through a Data Lens: The Case of China

**DOI:** 10.3390/ijerph17072309

**Published:** 2020-03-30

**Authors:** Jinling Hua, Rajib Shaw

**Affiliations:** Keio University, Fujisawa 252-0082, Japan; hana@sfc.keio.ac.jp

**Keywords:** COVID-19, Coronavirus, infodemic, humanitarian emergency, data science, good governance, citizen participation

## Abstract

Coronavirus (COVID-19) is a humanitarian emergency, which started in Wuhan in China in early December 2019, brought into the notice of the authorities in late December, early January 2020, and, after investigation, was declared as an emergency in the third week of January 2020. The WHO declared this as Public Health Emergency of International Concern (PHEIC) on 31th of January 2020, and finally a pandemic on 11th March 2020. As of March 24th, 2020, the virus has caused a casualty of over 16,600 people worldwide with more than 380,000 people confirmed as infected by it, of which more than 10,000 cases are serious. Mainly based on Chinese newspapers, social media and other digital platform data, this paper analyzes the timeline of the key actions taken by the government and people over three months in five different phases. It found that although there was an initial delay in responding, a unique combination of strong governance, strict regulation, strong community vigilance and citizen participation, and wise use of big data and digital technologies, were some of the key factors in China’s efforts to combat this virus. Being inviable and non-measurable (unlike radioactive exposure), appropriate and timely information is very important to form the basic foundation of mitigation and curative measures. Infodemic, as it is termed by WHO, is a key word, where different stakeholder’s participation, along with stricter regulation, is required to reduce the impact of fake news in this information age and social media. Although different countries will need different approaches, focusing on its humanitarian nature and addressing infodemic issues are the two critical factors for future global mitigation efforts.

## 1. Introduction 

Coronavirus (COVID-19) started spreading in December 2019 and was noticed in early January 2020. It started spreading in China in mid- to late-January. Among the different types of confusion and information challenges, we need to recognize that COVID-19 is first and foremost a humanitarian challenge [1]. As of 24 March, 2020, the virus has caused the death of over 16,600 people worldwide with more than 380,000 people are confirmed as infected by it, of which more than 10,000 are serious. As many as 184 out of 195 countries are affected. Solving the humanitarian challenge is the key priority through proper preventive measures to stop its spread, as well as curative measure to develop a vaccine. The impact of this public health emergency has affected countries and communities in terms of economic, socio-psychological issues, as well as international relations. 

“We’re not just fighting an epidemic; we’re fighting an infodemic”, said WHO Director–General Tedros Adhanom Ghebreyesus at the Munich Security Conference on 15 February 2020. WHO Information Network for Epidemics (EPI-WIN) was launched as a new information platform after WHO declared COVID-19 as a Public Health Emergency of International Concern (PHEIC). The goal was to share customized information with specific target groups [2]. Finally, on 11th March, WHO declared it this as a pandemic. 

“We know that every outbreak will be accompanied by a kind of tsunami of information, but also within this information you always have misinformation, rumors, etc. We know that even in the Middle Ages there was this phenomenon. “But the difference now with social media is that this phenomenon is amplified, it goes faster and further, like the viruses that travel with people and go faster and further. So it is a new challenge, and the challenge is the [timing] because you need to be faster if you want to fill the void...What is at stake during an outbreak is making sure people will do the right thing to control the disease or to mitigate its impact. So it is not only information to make sure people are informed; it is also making sure people are informed to act appropriately.” Said Sylvie Briand, Director of Infectious Hazards Management at WHO’s Health Emergencies Program and architect of WHO’s strategy to counter the infodemic risk. This poses the real challenge of mitigating the risk occurring from Coronavirus. 

One of the key issues of the “invisible disaster” is obtaining correct information. In 2011, Japan had a triple disaster, caused by an earthquake-induced tsunami, which caused a nuclear meltdown. A that time, there was a severe panic in and around Japan about the level of radiation, which was also an invisible disaster. However, radiation could be measured, whereas the level of penetration of the virus is not measurable. Therefore, providing the right information from a reliable source is the key issue in this type of pandemic.

Keeping this infodemic challenge in mind, this paper tries to analyze three months of happenings in China from December 2019 to February 2020, drawing and analyzing data from different Chinese websites, social media and research institutes. The value addition of this paper lies in the fact that original data were collected and analyzed in Chinese, and from Chinese social media. Although a characteristic information censorship exists in China, there were several positive and negative things that happened in the last three months. This paper is a narrative of those events and provides an original analysis.

There are three characteristics/impacts of the paper: (1) this is possibly the first analytical paper which uses firsthand social media and internet data and information from China to describe the time-series narrative in Wuhan and China with a focus on key policy decision, (2) it also uses original survey raw data to understand the types of media people used to get information, and (3) the reliance of different types of online services at different phases of the lockdown.

Of course, the paper has its own limitation, since, due to the evolving nature of the pandemic, the paper analyzes the spread in the original hotspot (although, as of late March 2020, the hotspot has shifted to Europe), which was Wuhan and the Hubei province of China. However, the key findings, which are described in Section 5, are useful to other parts of the world, which is currently suffering the impacts of COVID-19, as well as in future pandemic responses.

## 2. Characteristic of COVID-19 

The data on Coronavirus are changing on daily basis, and it is difficult to provide current statistics for the affected, recovered and casualties. However, based on some initial studies, a few characteristics are emerging for this virus. It is reported that the case-fatality-rate (CFR) for Coronavirus was 2.3%, initially; however, the age group of 70 to 79 has an 8% CFR, and CFR is 14.6% for those more than 80 years old [3]. This means that the virus has a stronger impact on the aged population.

The other characteristic of the virus is its speed in spreading. When Dr. Zhong Nan Shan made a public announcement of this virus in CCTV on the 20th of January, the virus had already spread in different provinces in China, as well as outside China. Every day, some new countries are added to the list, which has already reached more than 100 countries and regions. It took only 30 days to spread from one city to the entire country of China. The early cases may have been spread from the Wuhan seafood market, while later cases were spread from person to person, the speed of which surprised the health workers in Wuhan city and Hubei province. The epidemic curve shown in [4] as well as presented later in this paper, shows that the second to the third week of January was the most crucial time, when the spread was very high. 

There are some similarities and differences among COVID-19, Severe Acute Respiratory Syndrome: 2002–2003 (SARS) and Middle East Respiratory Syndrome: 2012-ongoing (MARS). SARS also had a zoonotic transmission in markets in Guangdong Province, China. It is said that COVID-19 is likely to have been transmitted from bats via palm civets. Similarly, MERS was also traced to zoonotic transmission of a novel coronavirus (likely from bats via dromedary camels) in Saudi Arabia. All three viruses have similar syndromes like fever and cough, which frequently lead to lower respiratory tract disease. However, SARS has a higher CFR of 9.6%, while MARS is even higher at a rate of 34.4%. Despite much higher CFRs for SARS and MERS, COVID-19 has led to more total deaths due to the large number of cases.

Projection shows a significant recession in the global economy due to Coronavirus spread [1]. The global surge reflects a new inflection point in this epidemic. Four ‘major transmission complexes’ (i.e., China, East Asia, Middle East, Europe) are now active, while the US is already at a tipping point. The analysis says that continued spread within established complexes plus community transmission in new complexes drives a ~0.3%–0.7% reduction in 2020 global GDP growth. The impact on demand slows down the growth of the global economy by between 1.8%–2.2%, instead of the 2.5% growth envisioned at the start of the year. Sectors are impacted differently. Certain sectors (e.g., aviation, tourism, hospitality) see lower demand for a longer duration. For others (e.g., consumer goods), demand is initially lower but expected to rebound quickly. The report also argues that 24th of February 2020 was a turning point, when the cases outside Chine exceeded in-China cases for the first time. South Korea, Italy, Iran, Japan and Singapore are the top five countries outside China which have reported a maximum number of cases, with Iran reporting the largest number of casualties outside China. 

## 3. Data Source and Methodology 

To focus on the key word “information”, which is crucial for any invisible disaster, a series of different types of data were analyzed. Primary data sources include: 

(1) Sina Weibo’s (Chinese social media) hot search list (in which a key word has been accessed every day for how many times as well as how many hours) [5]

(2) Corona Virus timeline data in China (which are compiled by the authors from different data sources like Sina, Tiki-Toki, Caixin, Baidu, Tencent and provincial and municipal government data) [6,7,8,9,10,11,12,13,14,15,16,17,18,19,20,21]

(3) CSM media research data on the use of different types of media to acquire information [22,23]; and

(4) Mob-Tech Research Institute data of use of internet during the Corona virus spread [24,25]. 

**Figure 1:**
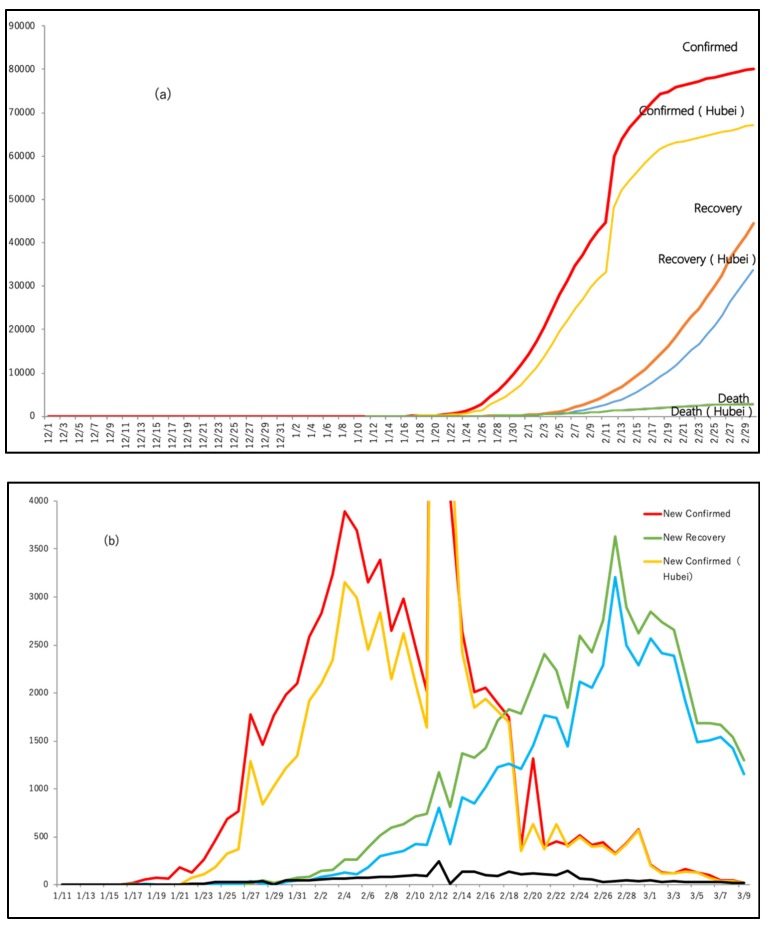
(**a**) Number of people affected, recovered, and dead in China and Hubei province; (**b**) New confirmed and recovered cases in China and Hubei province; (Source: these graphs were prepared by the authors using original data from: Sina News [5]).

**Figure 2:**
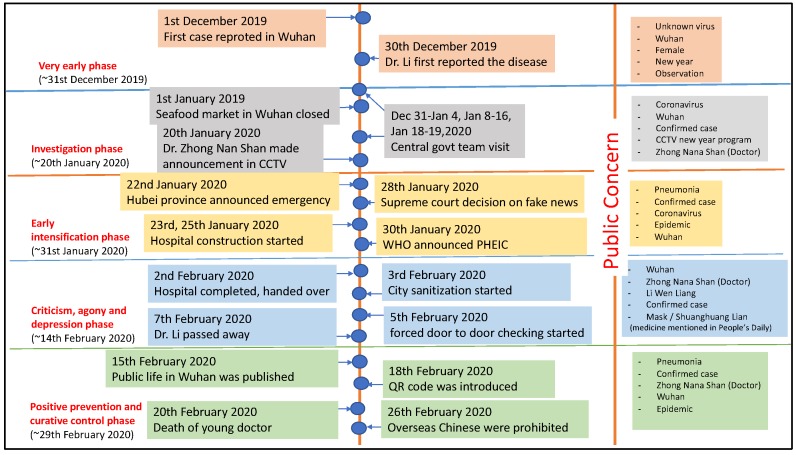
Timeline of key events and public concern; (Source: This figure was prepared by the authors using original data from: Sina, Tiki-Toki, Caixin, Baidu: [6,7,8,9,10,11,12,13,14,15,16,17,18,19,20,21]).

Four specific types of analysis were made based on the above-mentioned data sources:

(a) **Timeline narrative, number of affected people and public concern**: The timeline narrative is developed based on the sequential events in the country, and important measures taken, which is also juxtaposed to the major public concerns. Weibo’s data (2020) have been analyzed for the top 206 to 360 hits per topic (depends on the daily variation) over a period of three months, from 1 December 2019 to 1 March 2020 [24]. Tiki-Toki’s data [9] is the Chinese government big data platform, and provides information on different government measures, news, policies, and also is linked to major global milestones in the related topic (here, Coronavirus-related topics). Sina’s data was on (1) the number of affected people (confirmed cases, recovery, and death) at both the country level and in Hubei province, (2) Sina Weibo data to analyze social media information. Figure 1a,b show the growth in the number of affected and recovered people and casualties in Hubei province and the whole of China, which is referred to in a later section. Figure 2 was also prepared as an original diagram to highlight different phases of this disaster. Specific attention was made on the day to day changes in numbers, any significant policy actions taken, and any significant incidence (positive or negative) reported on social media or a website. Social media/website information (both government official sites as well private sites) were used to draw the timeseries narrative. Word Cloud analysis was made using the key words used in social media for all the five phases mentioned in the text, and the top five most commonly used words are picked to highlight the key discussion in the social media, as well as to understand citizens’ agony.
(b) **Media use during/after Coronavirus spread and information types:** This is mainly derived from the analysis of [23] on February 20–21, 2020, with more than 1500 residents from all 31 provinces in China, to understand the use of media to acquire information related to Corona virus. The analysis used the data of CSM survey to draw original graphs and diagrams with its interpretation;
(c) **Positive impact on certain online industries**: The data from [24] are based on the analysis of 2019 and 2020 analysis, but more specific intensive analysis of the use of the internet during the period of 22 January 2020 to 6 February 2020. These data were used to understand the proliferation of certain online services compared to others, which is correlated to people’s interest in different topics available online.

## 4. Data Analysis and Key Findings 

### 4.1. Narrative on the Events and Its Response Sequence 

To develop this narrative, as mentioned above, a large number of sources were consulted, reviewed and some milestones events are presented here. Needless to say, with a vast country like China, with the level of infection of Corona Virus, there are many small yet important events, which may be missed out here. However, the author tried to highlight the key developments in China based on the five following phases:

1. Very early phase: Until 31 December 2019;
2. Investigation phase: Until 20 January 2020;
3. Early intensification phase: Until 31 January 2020;
4. Criticism, agony and depression phase: Until 14 February 2020;
5. Positive prevention and curative control phase: Until 29 February 2020.

Figure 2 shows the timeline of key events in five phases. Authors extracted the key events from different news and social media reports. In each phase, five top public concerns are highlighted, which is prepared through word cloud analysis in each phase using the key social media data, as specified in the methodology section. 

#### 4.1.1. Very Early Phase: Until 31 December, 2019

As per the available statistics, the earliest case was reported on 1 December, 2019 in Wuhan, and thereafter sporadic cases have been reported all through December, especially in the later part of the month. The first case was reported in a paper [26] on 1st of December 2019. The health commission of Wuhan municipality reported the first case of Coronavirus (at the time, an unusual disease). These were unusual cases, which took the local physicians by surprise, and it was Dr. Li Wen Liang who reported the unusual case as a possible epidemic in WeChat social media on 30th of December 2019. 

#### 4.1.2. Investigation Phase: Until 20 January 2020

This phase was characterized by a crackdown by local government and detailed investigation. Huanan seafood market in Wuhan city was closed on 1st of January. The city government and its health commission investigated the cases in December and called Dr. Li and made him apologize for spreading a rumor on the 31st of December 2019. Three teams of experts from Beijing conducted a detailed investigation from the 31st December to 4th January, 8–16 January and 18–19 January. It was revealed that the disease was a new type of epidemic, which had not been reported earlier. This was announced on 20th of January by a major and well-known doctor, Dr. Zhong Nan Shan, in a CCTV online interview. 

#### 4.1.3. Early Intensification Phase: Until 31 January 2020 

This was a critical period, when the disease spread was intensified and a relatively large number of casualties was observed. Figure 1a shows the number of people affected, recovered and dead in China and Hubei province, and Figure 1b shows the new confirmed and recovered cases in China and Hubei province. At an early part of this phase, a few critical and wise decisions were made: 

22nd January: Hubei province announced a Level II public emergency;

23rd of January: Wuhan city was closed and all the entries and exits to the city were restricted. The decision to construct Huoshenshan Hospital (new hospital) for Corona virus cases was announced on this day (23rd January), followed the decision to construct Leishenshan Hospital (another new hospital) decision on 25th January. Ten hospitals in Wuhan city appealed for a supply of medical and other emergency goods from all over the country;

24th January: Hubei province followed the suit, and the whole province was closed for entrance and exit. Hubei, Beijing, Shanghai and eight other provinces declared a public emergency;

25th January: The Supreme court provided instruction on “Fake news” and the negative consequences of this. Tencent, which is the parent company of WeChat, established a website called “Rumors exposed website,” as a platform to reduce rumors;

26th of January: The first emergency supply arrived from Sichuan to Wuhan, along with medical and healthcare staff;

28th January: President Xi Xinping met WHO DG Dr. Tedros Adhanom Ghebreyesus and discussed the situation. China Media administration instructed all TV channels to reduce entertainment programs, and to increase broadcasting information and programs on Coronavirus and related news;

29th January: A countrywide emergency was declared;

30th January: The Emergency Committee on the novel coronavirus (2019-nCoV) under the International Health Regulations (IHR 2005) was reconvened by the World Health Organization Director–General Dr Tedros Adhanom Ghebreyesus on 30th January (Geneva time) and a Public Health Emergency of International Concern (PHEIC) was declared;

31st January: People’s Daily, the major Chinese newspaper’ official account, published fake news on a possible medicine (named Shuang Huang Lian, a Chinese antibiotic, of which online orders and users have drastically increased) for Coronavirus by mistake, which caused the panic-buying of the medicine by the public. 

#### 4.1.4. Criticism, Agony, Depression and Control Phase: Until 14 February 2020

The next phase was a phase of panic, criticism, agony and sad news. The following events took place that explain this phase:

31st January: Public criticism started on Chinese social media regarding the outbreak of the virus;

1st February: People’s Daily corrected their mistake regarding the fake news. A major media site, Caixin data analysis, showed that public agony had increased and people were growing worried about the future spread of the virus;

2nd February: The new hospital was prepared and handed over to the Army to take control;

3rd February: Sanitization of public spaces started, school entrance examinations were cancelled, and another new hospital was ready;

4–6th February: This was a time of control, where a few major control measures were taken. like a lockdown of villages, towns and cities (earlier, this was restricted to urban areas only). A new policy of “no one will be spared” was started (this enabled the government to enter people’s house and check for virus symptoms). Dou Ban, a major media group, was shut down. Overseas news, especially the spread of Coronavirus in a cruise ship in Japan (Diamond Princess) was broadcast in China through different media;

7th February: The first whistleblower from Wuhan, Dr. Li, passed away, and this caused severe public criticism in social media. This was followed by a depression phase, where several suicides by the infected people were observed, to save their respective family members;

9th February: The Center of Disease Control (CDC) head gave an online interview with Caixin and announced that the virus is a totally new type, of which not much is known yet. 

There were several incidences of sacking senior administration people; the China News head was sacked for spreading wrong information (12th February) and Wuhan’s Mayor was replaced (13th February). Holidays (school as well as offices) were extended. 

#### 4.1.5. Positive Prevention and Curative Control Phase: Until 29 February 2020

**Figure 3:**
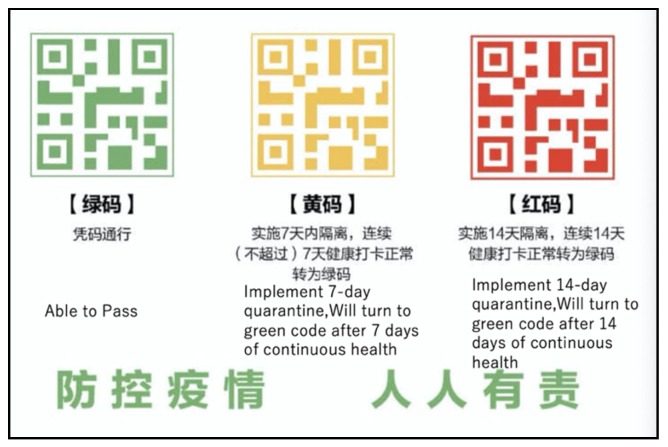
Three color codes used for monitoring people’s movement (Source: https://www.sina.com.cn [5]).

The following phase was a time of positive prevention and curative planning. Several new initiatives were taken through media to address public criticism as well as to lessen public agony:

15th February: A diary of public life in Wuhan was broadcast and shared through social media;

18th February: A touching story of female nurses cutting their hair to cope with the continuous work with protective suits was broadcast in the mass media as well as on social media. Dead bodies post-mortem had started to identify the key medical factors and impacts on the body. On the 18th of February, a unique approach of using a QR code was adopted in Wuhan and then spread to other parts of Hubei province using mobility and safety of the person (in terms of effect of Corona virus). This QR code was used for public transport, entering public areas. Using big data in mobile phones, three color coding were used (Figure 3): green (safe), yellow (need to be cautious), and red (cannot enter). Printed QR codes were used for the people who did not have mobile phones (like elderly people or children). On 19^th^ of February, the “no one will be spared” policy was ended. 

20th February: A newlywed young doctor passed away, which also created negative sentiment in social media. The issues of vulnerable people like the aged population (11 of them died in an old people’s home in Wuhan, along with the caregiver, which came out in the news on 20th February), physically and mentally challenged people, and their caregivers, received attention in the media;

21st and 22nd February: Data management and its authentication was re-ensured on (Jingzhou city), and goods distribution was re-investigated to ensure a balanced distribution (after a TikTok video which pointed out the imbalance in some areas). Punitive measures were taken for the two leaders of Hubei province for hiding information (22nd February);

23rd February: The last half of this phase was marked by mixed measures: ensuring the free flow of emergency goods and food and punishing those prohibited them (23rd February), and the death of two additional health professionals (23rd February);

26th February: New infection due to the return of overseas Chinese people in some selected provinces;

28th February: The unfortunate incidence of drinking sanitization tablet by mistake by some rural people, caused health issues, and there was another unfortunate incidence of suicide of a junior high school kid who did not have a mobile phone to undertake online classes provided by schools. 

### 4.2. Media Use during/after Coronavirus Spread and Information Types

**Figure 4:**
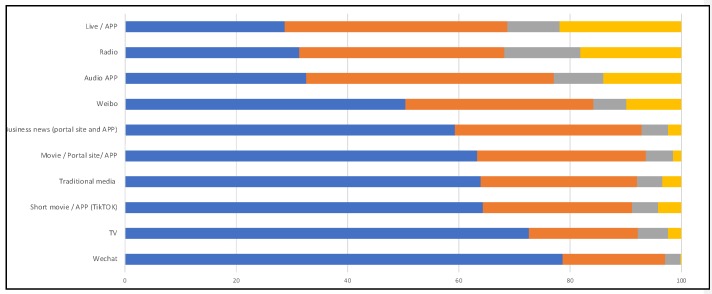
Use of different types of media before and after the Coronavirus spread; (Source: this figure was prepared by the authors using original data from: CSM Media Research [23]).

As mentioned above, an analysis was done by CSM Media Research with 1500 residents all over China on the mobile they use to access different sites. Figure 4 shows different media usage on four different aspects: increased usage after Coronavirus spread, same use as before, less use than before and do not use it for 6 months. Analysis shows that WeChat and TV played a strong role in acquiring information after Coronavirus spread. The amount of applications has also increased compared to other media usage, since it provides real-time information. 

The survey also pointed out that 44% people sought to proactively secure information, followed the news and put in their favorites. A total of 33% viewed the information proactively, but did not put it into their favorite news items. A total of 19% people saw the information if it was in the news or media; 2% of people did not bother to take any additional actions for information gathering, while another 2% did not want to hear the negative news on Coronavirus. 

**Figure 5:**
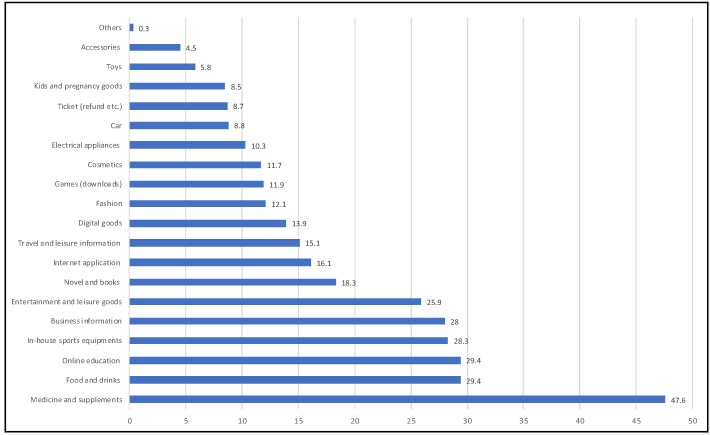
Types of information needed by people through online platforms; (Source: this figure was prepared by the authors using original data from: CSM Media Research [23]).

Figure 5 shows the types of information accessed by different users through online platforms. It shows that the maximum access was to get information on medicines, followed by a set of other information like food/drink, online education, in-house sports, business information and entertainment and leisure goods. This shows the lifestyle requirements when people were isolated in their home for a long period of time. 

### 4.3. Positive Impact on Certain Online Industries

As seen in Figure 5, medicine, food supply and online education were the top searched items. Online food supply users have drastically increased by 10 million, with an increase of 10.60 million in delivery capacity (by volume) when comparing the data of online food shopping of January 2019 with that of January 2020. A significant increase is observed in users with the age group 35 to 44 years, from 27.9% (January 2019) to 45.1% (January 2020). Specific increase has been noted with households with children from 0 to 3 years old (from 7.2 % of January 2019 to 25.2 % in January 2020). The available data show that four major online food supply companies have increased their delivery capacity by a significant percentage. They are as follows: Hema Fresh (up by 50% compared to before the virus spread), Miss Fresh (up by 321% compared to January 2019), Dingdong Maicai (up by 300% from December 2019), and Jing dong Daojia (up by 470% compared to January 2019). All the companies are struggling with a lack of human resources and shared their employees to help each other, which delayed delivery in several cases. 

Online education has also seen significant changes. On 27 January 2020, the Central Government Education Ministry has declared to postpone the start of classes until after the spring vacation. There was an instant rise in online education (Xueersi online internet school) after that, which saw a drastic increase by twenty-fold from 0.52 million to 11.54 million users within a period of one week (28 January to 6 February, 2020). 

## 5. Key Learning and Postscripts 

Through the Susceptible-Exposed-Infectious-Removed (SEIR) model and AI, [27] found that the epidemic of China should peak by late February, showing a gradual decline by the end of April. A five-day delay in implementation would have increased the epidemic size in mainland China three-fold. Lifting the Hubei quarantine would lead to a second epidemic peak in Hubei province in mid-March and extend the epidemic to late April, a result corroborated by the machine learning prediction.

WHO, in a recent joint study with Chinese colleagues, has summarized four specific key lessons as follows [3]:

1. China has rolled out perhaps the most ambitious, agile and aggressive disease containment effort in history. Although initially quite aggressive, gradually, a science and risk-based approach was taken to tailor its implementation;
2. Achieving China’s exceptional coverage with adherence to these containment measures has only been possible due to the deep commitment of the Chinese people to collective action in the face of this common threat. At a community level, this is reflected in the remarkable solidarity of provinces and cities in support of the most vulnerable populations and communities;
3. China’s bold approach to contain the rapid spread of this new respiratory pathogen has changed the course of a rapidly escalating and deadly epidemic;
4. China is already, and rightfully, working to bolster its economy, reopen its schools and return to a more normal semblance of its society, even as it works to contain the remaining chains of COVID-19 transmission.

From our own analysis, it was observed that the success of China’s efforts in controlling the disease was a combination of strong governance, strict regulation and spontaneous community/citizen participation. Although it was a late response in terms of the local and provincial government at the initial stage, once the disease was confirmed as a new one, collective responses at the community, ward, city, province and national levels were significant. To keep this large a number of people confined in their homes for almost two months was not an easy decision in terms of both economic and socio-psychological aspects. China’s mobile network and big data system was able to create the QR code-screening of people, which can be considered a significant achievement. As mentioned in the earlier part of this paper, WHO DG has termed this virus spread as infodemic; having the right information was key to the success of mitigation measures. At an early stage, The Supreme Court’s directives on fake news were a very good step in this regard to reduce the spread of confusion and panic. The “Rumors exposed website” created by Tencent (the parent company of WeChat) helped to share information on fake news and rumors effectively. Whenever there was fake news published or some mismanagement happened with the emergency goods and food supplies, quick corrective measures were taken by the authorities. At the village level, local communities and volunteers worked hard to ensure the implementation of the mitigation measures to reduce the spread as well as to report confirmed or suspected cases. At an early stage, data management was an issue, but once the virus was confirmed and declared by the government, strict data management measures were put into place. In this case, strict corrective measures were ensured for the mismanagement of data. The current case needs a science based solutions with local action [28].

These lessons are also reflected in the WHO research roadmap [29], where eight specific research issues have been identified with a balance of medical diagnosis and community use. It also emphasized social science research in the outbreak response, where the WHO will establish a team that will be integrated within multidisciplinary research and operational platforms and will connect with existing and expanded global networks of social sciences. As per [30], governments will not be able to minimize deaths from coronavirus disease 2019 (COVID-19) and the economic impact of viral spread. Keeping mortality as low as possible will be the highest priority for individuals; hence governments must put measures in place to ameliorate the inevitable economic downturn. In our view, COVID-19 has developed into a pandemic, with small chains of transmission in many countries and large chains resulting in extensive spread in a few countries, such as Italy, Iran, South Korea, and Japan. Most countries are likely to have a spread of COVID-19, at least in the early stages, before any mitigation measures have an impact [30]. 

As we started the paper with two key words “humanitarian challenge” and infodemic, we would like to once again highlight that basic humanitarian principles need to be followed in this type of emergency. Of course, there are geo-political, economic and social consequences, which also need to be looked at. However, humanitarian issues need to prevail over other priorities. The second point is that Coronavirus is a non-measurable disaster, unlike other invisible disasters like radioactive emission. Therefore, having correct and timely information is crucial for stopping its spread, as well as in the curative prevention of this disease. These two factors, along with good governance and citizen participation, will hold the key to success in combatting Coronavirus in future.
